# Supplementary material for: Could Collected Chemical Parameters Be Utilized to Build Soft Sensors Capable of Predicting the Provenance, Vintages, and Price Points of New Zealand Pinot Noir Wines Simultaneously?
Source: Foods. 2023 Jan 9;12(2):323. doi: 10.3390/foods12020323 (PMC9857561; doi:10.3390/foods12020323)
Supplement: Supplementary file 1 [file foods-12-00323-s001.zip › foods-2076027-supplementary.pdf]

Supplementary Materials

Could Collected Chemical Parameters Be Utilized to Build Soft Sensors Capable of Predicting the Provenance, Vintages, and Price Points of New Zealand Pinot Noir Wines Simultaneously?

Pinot noir wines

One hundred seventeen bottles of commercially New Zealand wines from five main regions (Central Otago, Marlborough, Martinborough, Nelson and North Canterbury) were studied, with retail prices of the wines ranging from NZ $10 to NZ $80, and vintage years from 2011 to 2020. Among one hundred seventeen bottles of commercially New Zealand Pinot noir wines, there are total 39 samples of New Zealand Pinot noir wines and every samples contain 3 bottles of Pinot noir wines. It means every 3 bottles of Pinot noir wines are from same brand and same vintage years. Among individual sample, two bottles of New Zealand Pinot noir wines evaluated by 7 experts and one bottles of New Zealand Pinot noir wines evaluated by 13 novices. Because novices only evaluated astringency, bitterness, sourness, astringency and clarity in Pinot noir wines, these sensory attributes will be ignored in this study.

Chemical reagent

Folin-Ciocalteu reagent (Merck), vanillin assay (99%, ECP), gallic acid (98%, ACROS), catechin-hydrate (99.8%, Sigma Aldrich), methyl cellulose (1500cp, Sigma Aldrich), methanol (Merck), ϱ-(dimethylamino)cinamaldehyde (ϱ-DMACA) (Sigma Aldrich), HCl (37%, Thermo Fisher Scientific), glycerol (99%, Thermo Fisher Scientific), Na_2CO_3 (99.5%, ECP), Ammonium sulfate (100.1%, AnalaR NORMAPUR®).

Chemical measurement procedure

Colour measurement

Shimadzu 2550 with 0.2 cm path length cuvettes were used for colour measurements. The colour of red wine can be determined at absorbance 420nm (A_{420nm}) for yellow colour, absorbance 520nm (A_{520nm}) for red colour and absorbance 620nm (A_{620nm}) for blue colour. (Dobrei, Poiana, Sala, Ghita, & Gergen, 2010).

Total phenolics assay

The total phenolic contents (TP) of wines were determined using the Folin-Ciocalteu method. Briefly, an aliquot (1 mL) of diluted wines was placed in a 10 mL volumetric flask, containing 5 mL of distilled water and 0.5 mL of Folin-Ciocalteu's reagent. After 3 min, 1.5 mL solution of Na_2CO_3 (20g/L) was added and the total volume was made up to 10 mL with distilled water. Samples were stored for 60 min at room temperature in sealed flasks surrounded by aluminium foil, and the absorbance was measured against the blank (distilled water) at 750nm. Gallic acid was used as a standard for the construction of the calibration curve. The concentration of TP was expressed in mg/L as gallic acid equivalents (Ivanova et al., 2011).

Total flavanol assay

Total flavanol content was evaluated by the vanillin assay, in which one molecule of vanillin reacts with one molecule of flavanol to produce a red chromophore. The conversion was monitored as an increase in absorbance at 500 nm. One volume of sample diluted in methanol was mixed with 2.5 volumes of vanillin (1% in methanol) and 2.5 volumes of HCl (9M in methanol). The mixture was incubated for 20 min at 35 ^{o}C before analysis. For each sample, a blank was used in which the vanillin solution was replaced with methanol alone. A standard curve was constructed with catechin-hydrate (mg/L) (Tabart, Kevers, Pincemail, Defraigne, & Dommes, 2010).

Total flavan-3-ols assay

The concentration of total flavan-3-ols was measured using the ϱ-

(dimethylamino)cinamaldehyde (ϱ-DMACA) method (Ivanova, Stefova, & Chinnici, 2010). The contents of flavan-3-ols in the wines are expressed as catechin-hydrate equivalents (mg/L). An aliquot (1mL) of an appropriately diluted sample was added to a 10 mL volumetric flask followed by the addition of 3 drops of glycerol and 5 mL ϱ-DMACA reagent. The total volume was made up to 10 mL with methanol and after 7 min, the absorbance was read at 640nm against a methanol blank. The DMACA reagent was prepared immediately before use, and contained 1% (w/v) DMACA in a cold mixture of methanol and HCl (4:1).

Total anthocyanins assay

Wine samples were diluted with a solution consisting of 70/30/1(v/v/v) ethanol/water/HCl (concentrated) and the absorbance was measured at 540nm (Ivanova, Stefova, & Chinnici, 2010). Due to the lack of a malvidin-3-glucoside standard, the total anthocyanic content are expressed as malvidin-3-glucoside equivalents (mg/L) and calculated using the following equation

TA_{540nm} (mg/L) =A_{540nm}16.7d (Ivanova, Stefova, & Chinnici, 2010)

Where A_{540nm} is the absorbance at 540nm and d is the dilution.

Total tannins assay

The use of other polymers to precipitate tannins has also been utilized in the methyl cellulose precipitable tannin assay (MCP). Briefly 3 mL MCP solution (0.04% w/v, 1500 cP viscosity) was added into 0.25 ml wine sample. After 1 minute, 2 mL of a saturated solution of $(NH_4)_2SO_4$ and 4.75 mL distilled water are added. A control sample is also prepared, but with distilled water instead of MCP solution. After 10 minutes, the samples are centrifuged for 5 min, and the tannin content is obtained by comparing the $A_{280nm}^{control}$ and A_{280nm} treatment referenced to catechin-hydrate equivalents (mg/L) (Aleixandre-Tudo, Buica, Nieuwoudt, Aleixandre, & Du Toit, 2017).

Chemical age

To 0.1 mL of wine, 10 mL of 1 N HCl was added and the value of absorbance was measured at 520nm in a 10 mm cuvette after 60 minutes. And then, this sample was measure at 520nm (A_{520nm}^{HCl}) and 280nm (A_{280nm}^{HCl}). Chemical age=$A_{520nm}^{HCl}/A_{280nm}^{HCl}$ (Dobrei, Poiana, Sala, Ghita, & Gergen, 2010).

<u>pH measurement</u>

Edge pH meter has been applied to measure Pinot noir wines' pH.

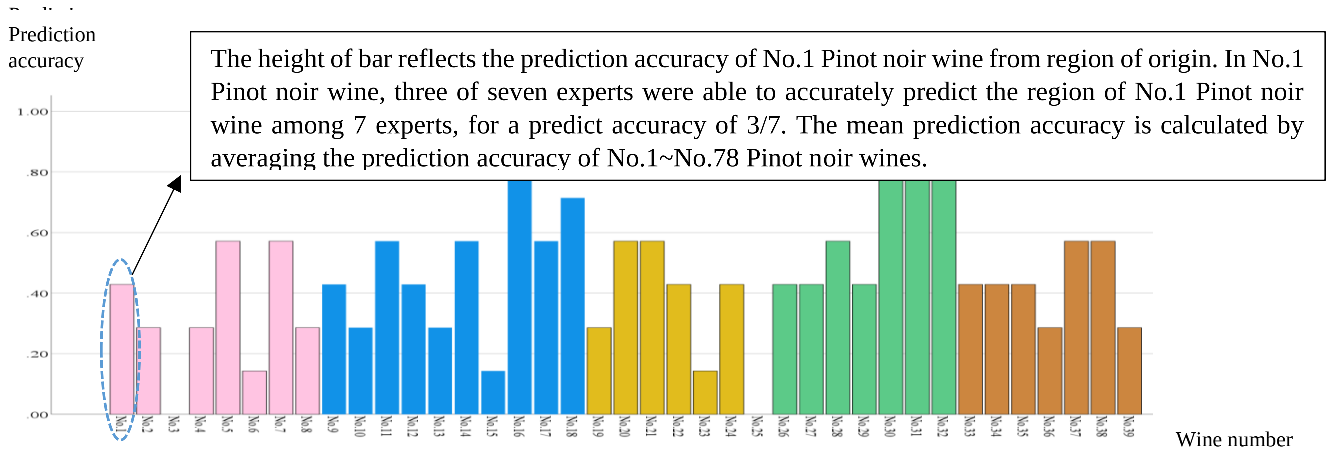

Prediction accuracy

(a) No.1-No.39 Pinot noir wines

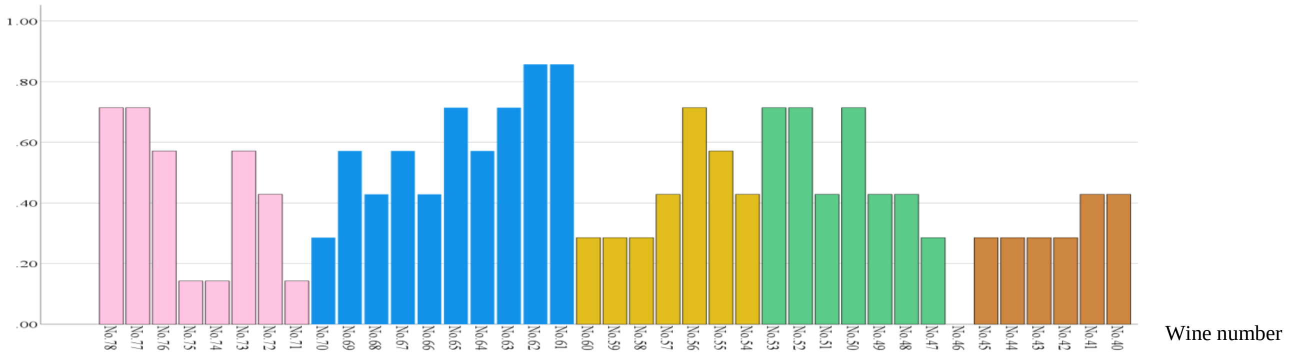

(b) No.40-No.78 Pinot noir wines

Figure S1. The prediction accuracy of experts on regions of origins. Figure S1 (a) displayed the prediction accuracies of No.1-No.39 Pinot noir wines evaluated by seven experts and Figure S1 (b) displayed the prediction accuracies of No.40-No.78 Pinot noir wines evaluated by seven experts.

Notes:

- Pink bar represents Pinot noir wines produced from Central Otago, blue bar represents Pinot noir wines produced from Marlborough, yellow bar represents Pinot noir wines produced from Nelson, green bar represents Pinot noir wines produced from Martinborough and bronze bar produced from North Canterbury.
- The prediction accuracy is calculated as follows, take No.1 Pinot noir wines as example, three of seven experts were able to accurately predict No.1 Pinot noir wines' regions of origin, the prediction accuracy is calculated as three divided seven.
- The mean accuracy is calculated the average prediction accuracy from No.1~No.78 Pinot noir wines with 45.421%.

Prediction
accuracy

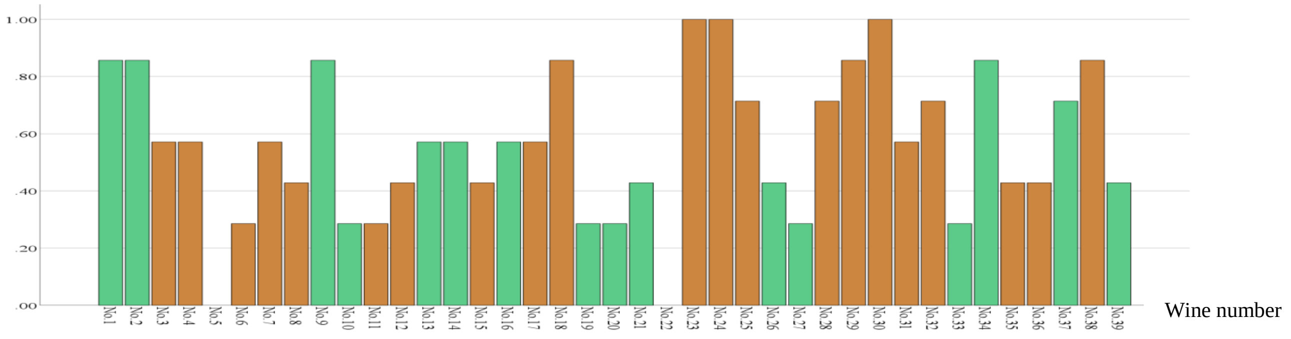

(a) No.1-No.39 Pinot noir wines

Prediction
accuracy

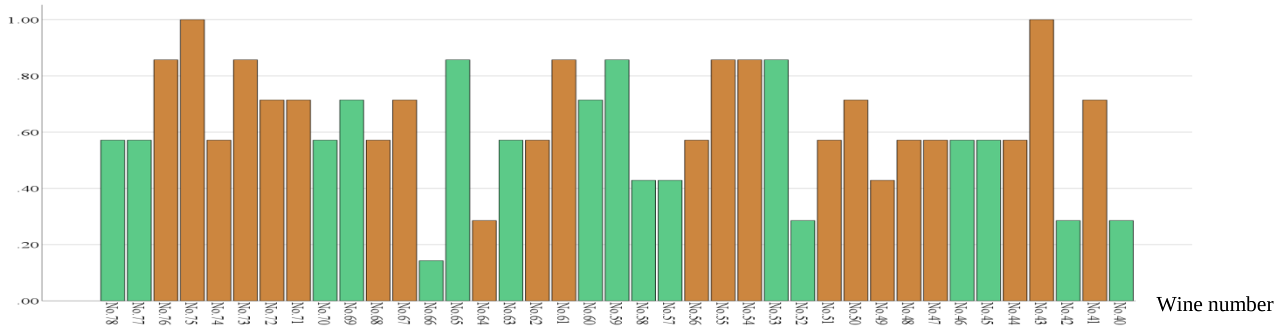

(b) No.40-No.78 Pinot noir wines

Figure S2. The prediction accuracy of experts on vintages. Figure S2 (a) displayed the prediction accuracies of No.1-No.39 Pinot noir wines evaluated by seven experts and Figure S2 (b) displayed the prediction accuracies of No.40-No.78 Pinot noir wines evaluated by seven experts.

Notes:

- Green bar represents Pinot noir wines with new vintages, bronze bar represents Pinot noir wines with old vintages.
- The prediction accuracy is calculated as follows, take No.1 Pinot noir wines as example, six of seven experts were able to accurately predict the vintage of Pinot noir wines, the prediction accuracy is calculated as six divided seven.
- The mean accuracy is calculated the average prediction accuracy from No.1~No.78 Pinot noir wines with 59.341%.

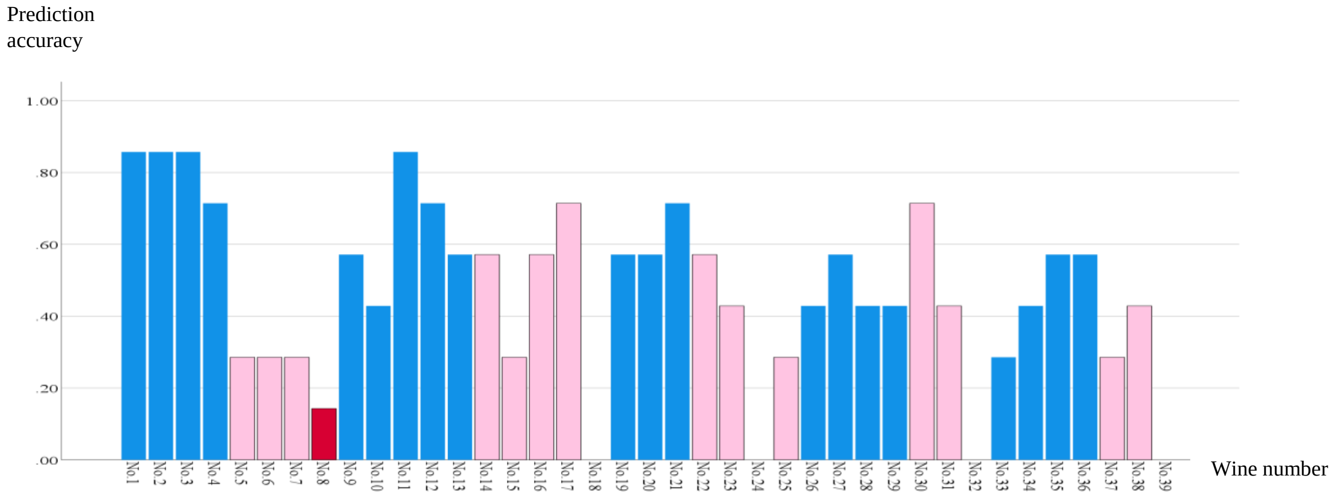

(a) No.1~No.39 Pinot noir wines

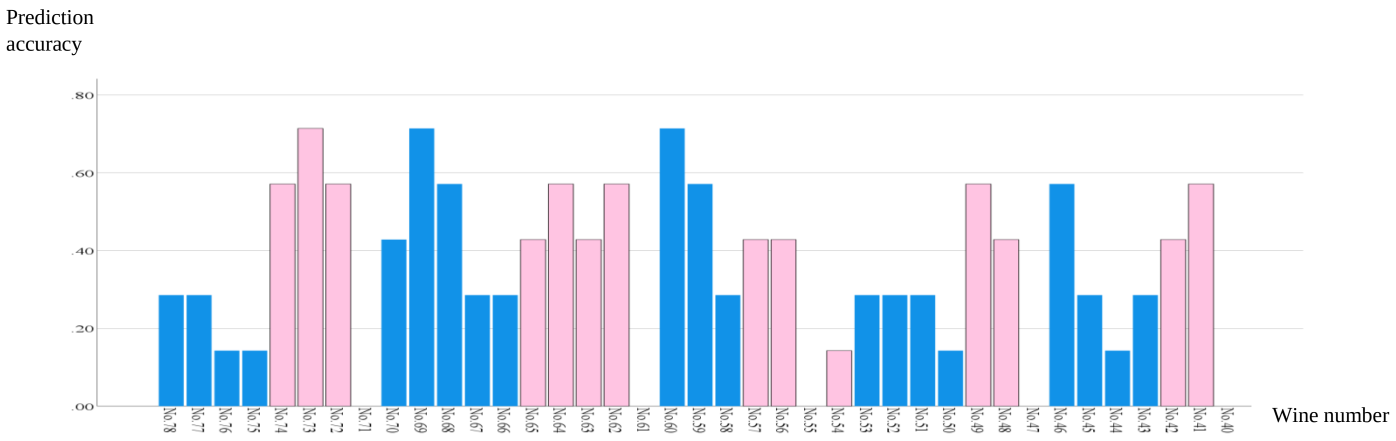

(b) No.40~No.78 Pinot noir wines

Figure S3. The prediction accuracy of experts on Price points. Figure S3 (a) displayed the prediction accuracies of No.1-No.39 Pinot noir wines evaluated by seven experts and Figure S3 (b) displayed the prediction accuracies of No.40-No.78 Pinot noir wines evaluated by seven experts.

Notes:

- Blue bar represents Pinot noir wines with low prices, pink bar represents Pinot noir wines with middle price and red bar represent Pinot noir wines with high prices.
- The prediction accuracy is calculated as follows, take No.1 Pinot noir wines as example, six of seven experts were able to accurately predict the price points of Pinot noir wines, the prediction accuracy is calculated as six divided seven.
- No.8 and No.71; No.18 and No.61, No.24 and No.55, No.32 and No.47, No.39 and No.40 are high prices.
  The mean accuracy is calculated the average prediction accuracy from No.1~No.78 Pinot noir wines with 41.209%.

During sensory evaluation, wine experts y were also required to anticipate the wines' regions of origin, vintages (old/new vintages), and price points. According to researchers, these Pinot noir wines are produced in five regions, including Central Otago, Marlborough, Nelson, Martinborough, and North Canterbury, with vintages ranging from 2011 to 2020 and prices between 10 and 80 New Zealand dollars. Once they had consumed one wine, they were expected to anticipate the regions of origin (Central Otago, Marlborough, Nelson, Martinborough, vintages (made before or after 2016), and prices (low, middle, or high prices). Taking No.1 Pinot noir wines as an example, three experts successfully forecasted Central Otago Pinot noir wines with an accuracy of 3/7 in Supplementary Figure 2 (a). The mean prediction accuracy was determined by averaging the prediction accuracy of the No. 1 to No. 78 Pinot noir wines.

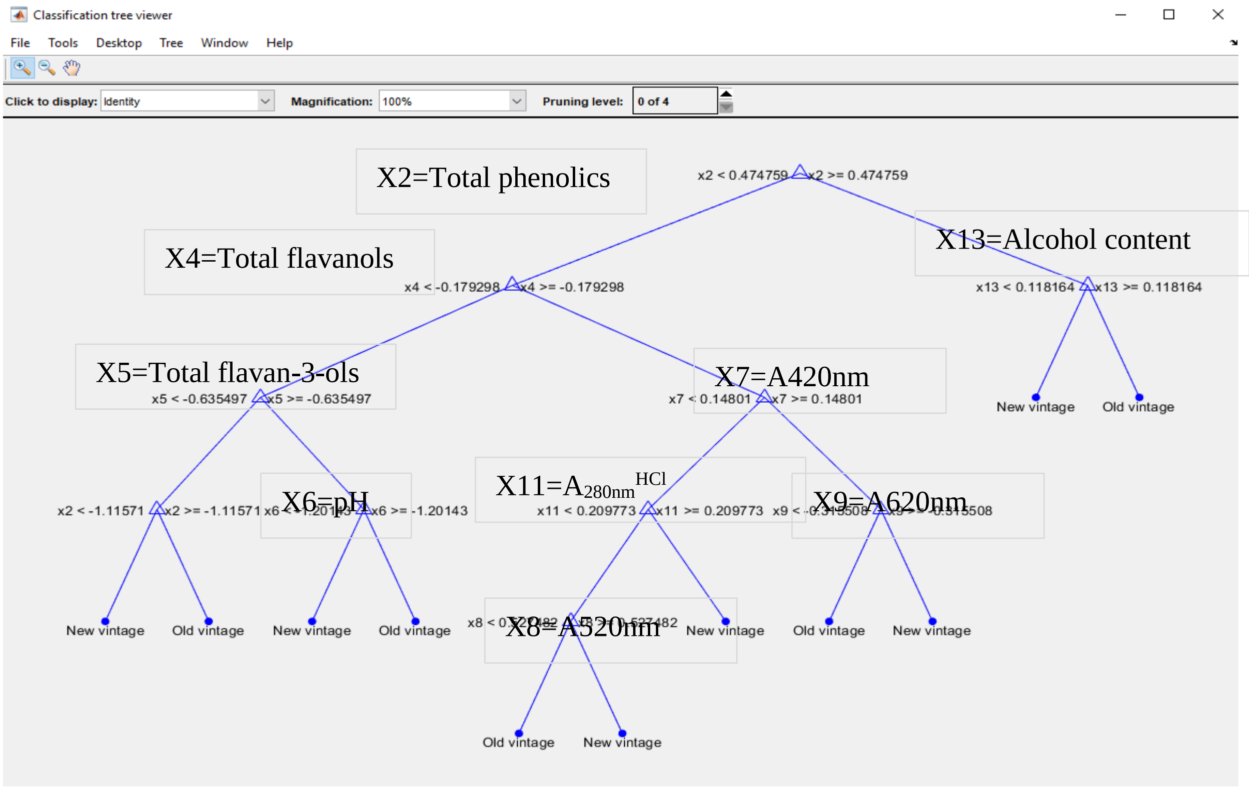

Figure S4. Classification tree structure about Pinot noir wines' vintages (old/new vintages)

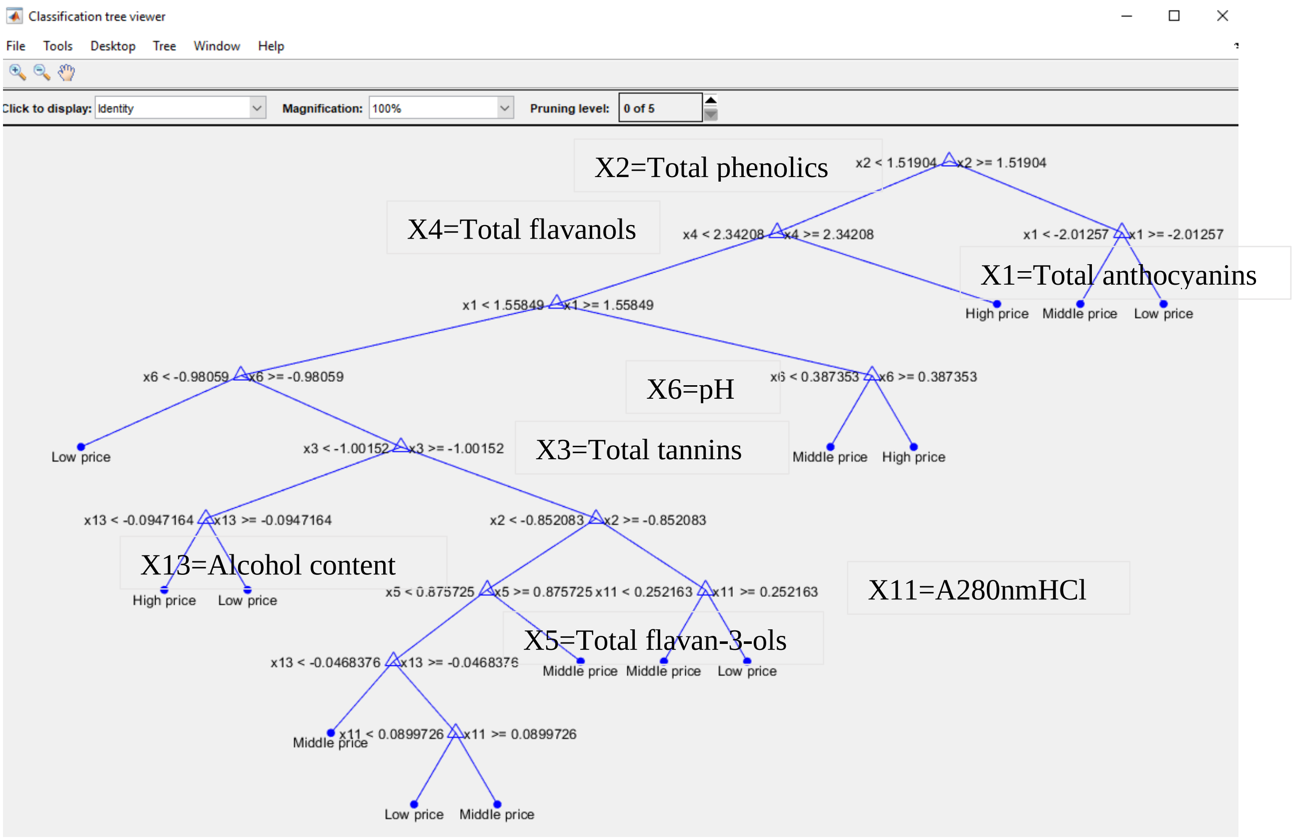

Figure S5. Classification tree structure about Pinot noir wines' price points

Table S1. Descriptive statistics of 117 bottles Pinot noir wines

	**Variables**	**Count**	**Mean**	**Standard deviation**
Phenolic variables (mg/L)	Total phenolics	117	3583	1077
	Total anthocyanins	117	105	51
	Total flavanols	117	992	408
	Total flavan-3ols	117	505	181
	Total tannins	117	936	432
Colour measurement (AU)	A_{420nm}	117	0.605	0.27
	A_{520nm}	117	0.607	0.266
	A_{620nm}	117	0.307	0.255
Chemical age measurement (AU)	A_{520nm}^{HCl}	117	0.161	0.082
	A_{280nm}^{HCl}	117	1.39	0.283
	Chemical age	117	0.116	0.054
Physicochemical variables	pH	117	3.66	0.127
	Alcohol content	117	0.133	0.005

Notes: AU indicates the absorbance at a Shimazdu 2550 spectrometer;

Table S2. 39 samples of New Zealand Pinot noir wines' product extrinsic cues (No.1-No.117 bottles)

Sample Number	Number	Region	Vintages	Price
Sample 1	No.1	Central Otago	2020	26
	No.78	Central Otago	2020	26
	No.79	Central Otago	2020	26
Sample 2	No.2	Central Otago	2019	27
	No.77	Central Otago	2019	27
	No.80	Central Otago	2019	27
Sample 3	No.3	Central Otago	2016	27
	No.76	Central Otago	2016	27
	No.81	Central Otago	2016	27
Sample 4	No.4	Central Otago	2014	23
	No.75	Central Otago	2014	23
	No.82	Central Otago	2014	23
Sample 5	No.5	Central Otago	2015	33

	No.74	Central Otago	2015	33
	No.83	Central Otago	2015	33
Sample 6	No.6	Central Otago	2018	33
	No.73	Central Otago	2018	33
	No.84	Central Otago	2018	33
Sample 7	No.7	Central Otago	2016	55
	No.72	Central Otago	2016	55
	No.85	Central Otago	2016	55
Sample 8	No.8	Central Otago	2014	70
	No.71	Central Otago	2014	70
	No.86	Central Otago	2014	70
Sample 9	No.9	Marlborough	2019	24
	No.70	Marlborough	2019	24
	No.87	Marlborough	2019	24
Sample 10	No.10	Marlborough	2018	16
	No.69	Marlborough	2018	16
	No.88	Marlborough	2018	16
Sample 11	No.11	Marlborough	2014	27
	No.68	Marlborough	2014	27
	No.89	Marlborough	2014	27
Sample 12	No.12	Marlborough	2012	27.5
	No.67	Marlborough	2012	27.5
	No.90	Marlborough	2012	27.5
Sample 13	No.13	Marlborough	2018	24
	No.66	Marlborough	2018	24
	No.91	Marlborough	2018	24
Sample 14	No.14	Marlborough	2017	48
	No.65	Marlborough	2017	48
	No.92	Marlborough	2017	48
Sample 15	No.15	Marlborough	2014	35
	No.64	Marlborough	2014	35
	No.93	Marlborough	2014	35
Sample 16	No.16	Marlborough	2017	35
	No.63	Marlborough	2017	35
	No.94	Marlborough	2017	35
Sample 17	No.17	Marlborough	2016	45
	No.62	Marlborough	2016	45
	No.95	Marlborough	2016	45
Sample 18	No.18	Marlborough	2013	63
	No.61	Marlborough	2013	63
	No.96	Marlborough	2013	63
Sample 19	No.19	Nelson	2018	14
	No.60	Nelson	2018	14

	No.97	Nelson	2018	14
Sample 20	No.20	Nelson	2019	22
	No.59	Nelson	2019	22
	No.98	Nelson	2019	22
Sample 21	No.21	Nelson	2017	20
	No.58	Nelson	2017	20
	No.99	Nelson	2017	20
Sample 22	No.22	Nelson	2017	40
	No.57	Nelson	2017	40
	No.100	Nelson	2017	40
Sample 23	No.23	Nelson	2015	39
	No.56	Nelson	2015	39
	No.101	Nelson	2015	39
Sample 24	No.24	Nelson	2016	75
	No.55	Nelson	2016	75
	No.102	Nelson	2016	75
Sample 25	No.25	Nelson	2015	50
	No.54	Nelson	2015	50
	No.103	Nelson	2015	50
Sample 26	No.26	Martinborough	2019	19
	No.53	Martinborough	2019	19
	No.104	Martinborough	2019	19
Sample 27	No.27	Martinborough	2018	24
	No.52	Martinborough	2018	24
	No.105	Martinborough	2018	24
Sample 28	No.28	Martinborough	2014	27
	No.51	Martinborough	2014	27
	No.106	Martinborough	2014	27
Sample 29	No.29	Martinborough	2011	27.5
	No.50	Martinborough	2011	27.5
	No.107	Martinborough	2011	27.5
Sample 30	No.30	Martinborough	2016	40
	No.49	Martinborough	2016	40
	No.108	Martinborough	2016	40
Sample 31	No.31	Martinborough	2014	30
	No.48	Martinborough	2014	30
	No.109	Martinborough	2014	30
Sample 32	No.32	Martinborough	2016	65
	No.47	Martinborough	2016	65
	No.110	Martinborough	2016	65
Sample 33	No.33	North Canterbury	2020	18
	No.46	North Canterbury	2020	18
	No.111	North Canterbury	2020	18

Sample 34	No.34	North Canterbury	2018	15
	No.45	North Canterbury	2018	15
	No.112	North Canterbury	2018	15
Sample 35	No.35	North Canterbury	2016	22
	No.44	North Canterbury	2016	22
	No.113	North Canterbury	2016	22
Sample 36	No.36	North Canterbury	2014	27
	No.43	North Canterbury	2014	27
	No.114	North Canterbury	2014	27
Sample 37	No.37	North Canterbury	2018	50
	No.42	North Canterbury	2018	50
	No.115	North Canterbury	2018	50
Sample 38	No.38	North Canterbury	2016	43
	No.41	North Canterbury	2016	43
	No.116	North Canterbury	2016	43
Sample 39	No.39	North Canterbury	2017	79
	No.40	North Canterbury	2017	79
	No.117	North Canterbury	2017	79
